# “On the same page”? The effect of GP examiner feedback on differences in rating severity in clinical assessments: a pre/post intervention study

**DOI:** 10.1186/s12909-017-0929-9

**Published:** 2017-06-06

**Authors:** Nancy Sturman, Remo Ostini, Wai Yee Wong, Jianzhen Zhang, Michael David

**Affiliations:** 1 Primary Care Clinical Unit, Faculty of Medicine, The University of Queensland, Brisbane, Queensland Australia; 2 Royal Brisbane & Women’s Hospitals, Level 8, Health Sciences Building, Herston, QLD Australia; 3 Rural Clinical School Research Centre, Faculty of Medicine, The University of Queensland, Brisbane, Queensland Australia; 4 Institute for Teaching and Learning Innovation, The University of Queensland, Brisbane, Queensland Australia; 5 Centre for Chronic Disease, Faculty of Medicine, The University of Queensland, Brisbane, Queensland Australia; 6 School of Public Health, The University of Queensland, Queensland, Australia

## Abstract

**Background:**

Robust and defensible clinical assessments attempt to minimise differences in student grades which are due to differences in examiner severity (stringency and leniency). Unfortunately there is little evidence to date that examiner training and feedback interventions are effective; “physician raters” have indeed been deemed “impervious to feedback”. Our aim was to investigate the effectiveness of a general practitioner examiner feedback intervention, and explore examiner attitudes to this.

**Methods:**

Sixteen examiners were provided with a written summary of all examiner ratings in medical student clinical case examinations over the preceding 18 months, enabling them to identify their own rating data and compare it with other examiners. Examiner ratings and examiner severity self-estimates were analysed pre and post intervention, using non-parametric bootstrapping, multivariable linear regression, intra-class correlation and Spearman’s correlation analyses. Examiners completed a survey exploring their perceptions of the usefulness and acceptability of the intervention, including what (if anything) examiners planned to do differently as a result of the feedback.

**Results:**

Examiner severity self-estimates were relatively poorly correlated with measured severity on the two clinical case examination types pre-intervention (0.29 and 0.67) and were less accurate post-intervention. No significant effect of the intervention was identified, when differences in case difficulty were controlled for, although there were fewer outlier examiners post-intervention. Drift in examiner severity over time prior to the intervention was observed. Participants rated the intervention as interesting and useful, and survey comments indicated that fairness, reassurance, and understanding examiner colleagues are important to examiners.

**Conclusions:**

Despite our participants being receptive to our feedback and wanting to be “on the same page”, we did not demonstrate effective use of the feedback to change their rating behaviours. Calibration of severity appears to be difficult for examiners, and further research into better ways of providing more effective feedback is indicated.

## Background

One long recognised challenge in clinical assessment is minimising differences in student grades which are due to inconsistencies in examiner ratings. Whether this challenge is addressed from a psychometric perspective, for example generalizability theory [1], or from social cognition frameworks [2–5], student grades should depend as little as possible on who examines them.

Inconsistencies in examiner ratings are related to a number of factors, including differences in examiner severity. “Examiner severity” is an examiner’s position on a spectrum from very lenient to very stringent, reflecting a consistent tendency to use a particular part of the relevant rating scale in their ratings [6]. Examiners with differences in severity assign different scores to examinees (although they may rank them and/or diagnose their strengths and weaknesses similarly). Several studies have shown that clinician examiners manifest different levels of severity, and that this has a significant impact on examinee grades and assessment decisions across a range of clinical skills assessments. These include work-based assessments, oral examinations and OSCEs [5–8]. Although extreme differences in examiner severity are probably relatively uncommon [9] modest differences in examiner severity may make important differences to student grades and pass-fail decisions.

Differences in examiner severity are poorly understood. Lenient examiners may tend to be “candidate–centred”, whereas stringent examiners may be more focused on maintaining high clinical standards [10]. Differences in severity may be linked to different examiner conceptions of standards, and of academic failure [11]. Severity may also be conceptualised as a type of sensitivity-specificity trade-off: more stringent examiners may prioritise detecting (and failing) incompetent students more highly (sensitivity) than avoiding failing competent ones (specificity), and indeed there is some evidence that sensitivity and specificity correspond to two different metacognitive processes associated with calibration [12]. Attempts to understand examiner severity are also complicated by the discrepancy that can exist between public ratings and private judgements about examinees, including a reluctance to fail underperforming students [2].

Little is also known about examiner perceptions of their own severity. A poor correlation (0.11) was found between family medicine OSCE examiners’ self-perceived severity and the severity of their ratings [7], and stringent examiners were unaware of their “hawkishness” in another study [9]. Little is known about the willingness and/or ability of examiners to change their severity after receiving feedback. Although Harasym et al. reported that “feedback to examiners on their stringency/leniency did not change their behaviour”, they included very little information about the nature of this feedback [7]. Studies dating from the 1970s have evaluated various examiner training and feedback strategies (including didactic teaching about cognitive error and biases, and rating practice using videotaped performances with feedback of expert ratings and facilitated discussion of inter-examiner differences) and found weak, if any, evidence of their effectiveness [13–15]. Several authors have speculated that “physician raters” might be “impervious to training”, and suggested that outlier examiners are best managed by excluding them from further participation in assessments [14] or using equating or adjusting strategies to compensate for severity differences [6, 16]. However it remains plausible that more effective examiner interventions may yet be identified.

In this study we investigated the effect of an examiner feedback intervention on the severity of examiner ratings. We provided general practitioner examiners with written feedback comparing all examiners’ ratings over the preceding 18 months of general practice clinical case examinations. The intervention was intended to assist examiners to “calibrate” their rating judgements with those of their examiner colleagues, and reduce differences in examiner severity.

The study was designed to test the following hypotheses:

1. Examiner self-perceptions of rating severity (“severity self-estimates”) will be more accurate following the intervention
2. Ratings of more stringent examiners will tend to become more lenient, and ratings of more lenient examiners will tend to become more stringent, following the intervention

We also wished to investigate the stability of examiner severity over time, and examiner perceptions of the acceptability and usefulness of the intervention.

## Methods

All examiners who had examined in any general practice clinical case examinations, in the 18 months prior to the intervention, were invited to participate in this study. In these examinations, third year University of Queensland medical students completing their general practice placements were examined by general practitioner examiners on two standardised clinical cases using standardised patients, a Diagnostic Case focused on diagnostic skills and a Management Case focused on patient management. Each case was rated using a standard marking rubric with four criteria rated from 1 (unsafe) to 7 (exemplary); these four item level ratings were averaged to give a final score out of seven on each case, and the passing standard was set at 4. Both the Diagnostic and Management Cases assessed students on communication skills. The Diagnostic Case also assessed students on history-taking, physical examination and formulating differential diagnoses. The Management Case also assessed students on the content of their management, consultation structure, and management of an ethical or professionalism issue. Examiners from the examiner pool examined in a variable number of sessions, working with various standardised patients, over the study period.

Examinations were conducted five times a year in Brisbane, Queensland. All examiners were practising general practitioners with active involvement in practice-based teaching and/or tutorial teaching. The examination cases were selected from a database of approximately forty recently developed standardised cases, and students were allocated 18 min to complete each case (including perusal time). Students were assigned to examiners predominantly on a consecutive alphabetical basis, although a small number of manual adjustments were made, for student convenience or to avoid examiners examining students who were particularly well known to them.

Prior to the intervention, the examiner participants provided basic demographic information, and indicated their previous examiner experience. They were invited to “rate yourself in terms of your leniency or stringency as an examiner in recent general practice clinical case examinations” on a visual analogue scale, from “very lenient” to “very stringent”.

The intervention was administered in July 2013, and consisted of the provision of a written summary of individual examiner ratings over the preceding 18 months (see Table 1). The same information was presented to all examiners, in a de-identified, coded format. Examiners were supplied only with their own code, in order to enable them to identify their own data but not identify other examiners in the data provided.

**Table 1:** Feedback to examiners on Case Ratings^a^

1	Overall mean rating, Standard Deviation (SD), and range of each examiner’s Diagnostic^b^ Case ratings
2	Mean differences between each examiner’s mean rating and the mean ratings of every other examiner, highlighting significant differences
3	Overall mean rating and SD for each Diagnostic^b^ case
4	Mean ratings, SDs, and range of ratings for each examiner on each Diagnostic^b^ case examined

^a^Information for the 2012 and 2013 examinations was presented separately

^b^The equivalent information was also presented for Management Cases

Examiners were invited to complete a survey exploring their perceptions of the usefulness and acceptability of the intervention feedback. The survey included free form responses to questions about what was learned from the feedback, which aspects were useful, and what (if anything) examiners planned to do differently as a result of the feedback.

Following the intervention, a further two examinations were conducted, after which participants were invited to complete a second retrospective severity self-estimate based on these most recent examinations.

To test Hypotheses 1, Spearman’s correlations between examiner severity self-estimates, and examiner mean overall ratings for the two examinations preceding these estimates, were performed, and pre- and post- intervention correlations were compared.

**Fig. 1:**
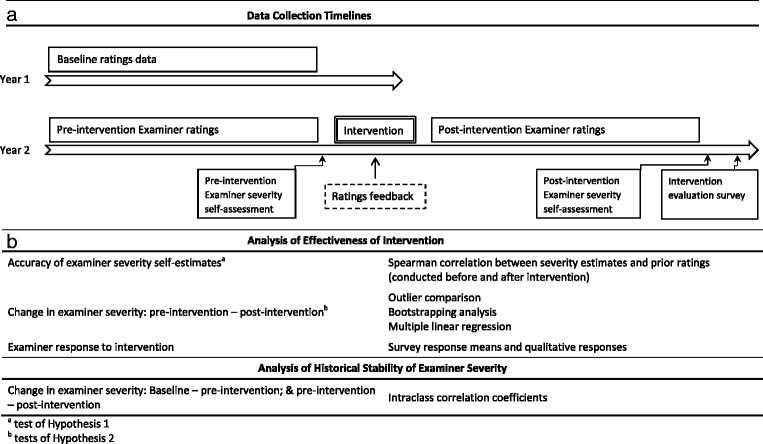
Data collection timelines (**a**) and analysis schema (**b**)

To test Hypothesis 2, we compared the number of outlier examiners (defined here as the number of examiners whose individual mean rating was more than three Standard Errors from the overall examiner mean rating) pre and post intervention. A non-parametric bootstrapping method using 10,000 iterations [17] was also used to compare the direction of change in examiner ratings following the intervention, between those examiners who were initially more lenient than the median examiner in their pre-intervention ratings and those examiners who were initially more stringent. A multivariable linear regression was also performed on average examiner ratings, controlling for differences in individual Case difficulty by including overall average Case rating as a variable. The variable of interest in this analysis was the change in examiner average ratings post-intervention. See Fig. 1 for a summary of study timelines, and analyses of effectiveness of intervention.

Intra-class coefficients were calculated to identify the historical relationship between 2012 and 2013 examiner ratings (prior to the intervention) and the relationship between examiner ratings pre and post-intervention.

Post-intervention survey data were analysed using descriptive statistics and a content analysis. Two investigators (NS and AW) independently coded and categorised the free form survey responses descriptively prior to meeting to identify emerging themes by consensus [18].

## Results

Sixteen of the seventeen examiners who examined during the study period consented to participate in the project; the non-consenting examiner ceased examining in August 2012. One consenting examiner did not return any survey responses. Participant demographic information and relevant experience are shown in Table 2.

**Table 2:** Demographics and Experience of General Practitioner Examiner Participants (*N* = 15)

Gender	Female 9: Male 6
General practice experience (range in years)	1.5–35
Medical student teaching experience (range in years)	0.5–33
Number of general practice clinical case examinations previously examined by examiner (range)	2–30
Previous participation in informal discussions about assessment with other examiners	Yes:15 No: 0
Previous participation in an examiner training session	Yes: 13 No: 2
Previous experience of co-marking with another examiner	Yes: 12 No: 3

A total of 240 ratings from the two examinations immediately pre-intervention and 240 ratings from the two examinations post-intervention were analysed; there were no missing ratings for these examinations. Participating examiners marked between 1 and 8 different cases, and between 8 and 60 individual students over these four examinations. Of the 15 participants who examined in these examinations in 2013, eight examined both pre- and post-intervention.

Summary descriptive statistical data for participating examiner ratings are presented in Table 3. The distribution of ratings approximated to a normal distribution.

**Table 3:** Examiner overall ratings

	Pre-Intervention *N* = 120	Post-Intervention *n* = 120
Diagnostic Case Mean	5.035	5.129
Diagnostic Case Standard Deviation	0.941	0.881
Diagnostic Case Range	3.00–7.00	2.5–7.00
Number of outlier examiners in Diagnostic Case Examinations^a^	8 (out of 11 examiners)	7 (out of 13 examiners)
Management Case Mean	4.874	4.990
Management Case Standard Deviation	1.104	0.954
Management Case Range	2.75–6.75	3.75–7.00
Number of outlier examiners in Management Case Examinations^a^	5 (out of 9 examiners)	4 (out of 13 examiners)

^a^Examiners whose mean rating was more than 3 standard errors from the overall mean

Participant severity self-estimates ranged from 3 to 7 on a visual analogue scale, from 1 (very lenient) to 10 (very stringent). Severity self-estimates had a median of 6 and a mean of 5.3. No participant demographic factors were significantly correlated with average overall ratings.

There was a weak or modest correlation between severity self-estimates and average overall ratings pre- intervention (Spearman correlation 0.29 for diagnostic case and 0.67 for management case). There was a negative correlation post-intervention (Spearman correlation −0.52 for diagnostic case and −0.80 for management case).

The number of outlier examiners was lower after the intervention than before the intervention, for both Diagnostic and Management Cases. The bootstrapping analysis showed a significant increase in the ratings of more stringent examiners post-intervention, and a significant difference between the direction of change in ratings post-intervention comparing stringent and lenient examiners. However this was found only for Diagnostic Cases, and the ratings of more lenient examiners did not decrease significantly for either Case type. No significant effect of the intervention for either Case type was found in the Multivariable Linear Regression analysis. The intra-class correlation analysis (see Table 4) found that intra-examiner ratings were more consistent pre and post intervention than they were historically between 2012 and 2013. These results are summarized in Table 4.

**Table 4:** Changes in examiner ratings

(a) Analysis of Effectiveness of Intervention		
Outlier Analysis		
	Diagnostic Case Type	Management Case Type
Pre-intervention	8 (out of 11 examiners)	5 (out of 9 examiners)
Post-intervention	7 (out of 13 examiners)	4 (out of 13 examiners)
Bootstrapping Analysis		
	Diagnostic Case Type	Management Case Type
Change in lenient examiner ratings	−0.18 (95%CI −0.52 – +0.17)	−0.18 (95%CI −0.79 – +0.34)
Change in stringent examiner ratings	+0.37 (95%CI +0.14 – +0.28)	+0.17 (95%CI −0.35 – +0.64)
Difference in change between lenient and stringent examiners	+0.55 (95%CI +0.05 – +0.68)	+0.35 (95% CI −0.37 – +1.07)
Multivariable Linear Regression		
	Diagnostic Case Type	Management Case Type
Full model	F (4,3) = 4.96; *p* > 0.11	F (5,1) = 0.52; *p* > 0.77
Intervention effect	t = 0.54; *p* > 0.62	t = −0.16; *p* > 0.90
(b) Analysis of Historical Stability of Examiner Severity		
Intra-class Correlation Analysis		
	Diagnostic Case Type	Management Case Type
Intra-examiner correlation between average ratings 2012 and pre-intervention 2013	0.420	0.179
Intra-examiner correlation between average ratings pre- and post-intervention 2013	0.665	0.578

Findings from the post-intervention survey (presented in Table 5) indicated that the marking feedback intervention was perceived as interesting and useful. The key emerging themes from the free text comments were fairness of examinations, reassurance for examiners, and understanding other examiners (see illustrative quotations in Table 5).

**Table 5:** Examiner attitudes to examiner feedback (*N* = 14)

*Survey question* *Rated from Strongly disagree(1) to Strongly agree(5)*	*Average score (range)*
The comparative examiner marking feedback was useful to me in informing me about my leniency or stringency as an examiner	4.3 (3–5)
The comparative examiner marking feedback was easy to understand	3.6 (1–5)
I am interested in receiving comparative examiner marking feedback in future	4.4 (3–5)
Comparative marking feedback is effective in improving the reliability of our examinations	4.0 (3–5)
*Survey comments on the usefulness and uses of the marking feedback* *Key themes and illustrative responses*	
*Fairness* “to ensure that we are on the same page and that students are being examined as fairly as possible” P3 “Helps to identify whether I am “in the ball park” or too extreme, as these are judgements and need to be calibrated” P8	
*Reassurance* “I thought I was being to (sic) lenient, but my mind has been put at ease” P14 “Also supports decisions to fail students which are hard, if I can see that other examiners are prepared to do this too” P8	
*Understanding* “(to) see what other examiners find important in the students’ performance and to get an understanding of what level the students are expected to be at” P14 “Comparing marks with other examiners helps align your marks with your peers, but also helps understand other examiners’ approach to exams and situations and examining” P4	

In answer to the survey question about any changes examiners planned to make to their marking as a result of the feedback, seven (out of fifteen) participants indicated that they did not plan to make any changes. Free text comments from two relatively lenient examiners who did plan to change included plans to “*be more aware generally of where I feel students should sit on the marking scale*” (P3) and “*be careful not to mark up or avoid poor marks without a clear reason*” (P12). A relatively stringent examiner planned to “*pay more strict attention to the marking criteria on the marking sheet*” (P11). Another relatively stringent examiner (P4) commented that the feedback “*accords with my self-belief that I am not particularly lenient*”, adding that “*I doubt that I will change my examining practice as a result of seeing where I sit*”. Another examiner (P9) commented that “*we may all come to mark more uniformly (or perhaps not) based on this information, but perhaps we need more information to know if this makes our marking any more valid.*”

## Discussion

We found no evidence that our examiner intervention, intended to reduce differences in examiner severity, was effective. This is in keeping with previous literature, in which well-intentioned examiner training and feedback has not proven effective. Significant differences in examiner severity were present both pre and post intervention, as shown by the number of examiners whose average rating was more than 3 standard errors from the overall mean (see Table 3). There did appear to be fewer of these outlier examiners post-intervention, but this is of uncertain significance. Our examiners were unable to estimate their own severity accurately, and indeed their severity self-estimates were less accurate after they were provided with our marking feedback. There are well-known methodological problems with research on the accuracy of self-assessment [19] including assumptions that participants are “measuring the same dimensions of performance using the scale in the same way”. The validity of using a visual analogue scale anchored from lenient to stringent for self-assessment of examiner severity could be contested. Indeed, the concept of examiner severity itself probably warrants further discussion in the literature. Our new finding of reduced self-assessment accuracy post-intervention should be tested in further research. A limitation of our study is that it was not possible to disentangle student ability, case difficulty, standardised patient factors, examiner severity, and their interactions. This was due to the disconnected design of our examinations, including the nesting of examiners in Cases, which precluded the use of a generalizability study [20]. We investigated differences in examiner severity using a number of other analyses, however.

We included survey data and free form responses which enabled us to explore examiner attitudes to the intervention. Our findings suggest that examiners were committed to fair and reliable examinations, and interested in receiving marking feedback and engaging in further discussion with other examiners. Some participants were cautious about using the marking feedback we provided to calibrate their rating behaviours, partly because they were open to the possibility that outlier examiners (including themselves) were making valid judgements. Other participants indicated that they would attempt to re-calibrate their severity, but appeared to be unsuccessful in this attempt.

We also found that examiner severity was unstable even in the absence of an examiner feedback intervention, particularly for our Management Cases. This inherent instability may complicate studies of examiner training strategies. Previous findings from investigations of intra-rater consistency in clinical assessment have been conflicting, and often have difficulty distinguishing drifts in examiner severity from drifts in task difficulty (even the same task may be easier for a better prepared examinee cohort, for example). Several studies have found that examiner severity was relatively stable over time and examinees, in clinical long cases [21] OSCE stations [10], short answer questions [22] and oral examinations [23]. However, in other studies severity indices for standardised patient and clinician OSCE examiners drifted substantially from their initial value after 3 or 4 months, particularly on generic rating scales of interpersonal and communication skills [24], and a small number of standardised patient examiners drifted significantly even within a three month time period [25]. Hemmer found that examiners became more stringent after group discussion with other examiners [26]. This instability merits further investigation, and suggests caution in using examiner equating or adjustment strategies based on previous examiner severity. The findings that severity may be more stable on some items, and for some examiners, than others, suggests that item-level and individual examiner-level analyses may further inform this area.

## Conclusion

Although investigations of examiner severity in authentic settings are difficult conceptually and methodologically, and no intervention to reduce differences in severity has been proven effective to date, it is generally agreed that defensible clinical assessments should minimise differences in student grades which are due to inconsistencies in examiner ratings, including differences in examiner severity. Although increasing the number of assessment nodes, contexts and/or examiners may even out these inconsistencies [1], it remains important to calibrate examiners as effectively as possible, especially if this increase is not feasible. Although our intervention did not appear to be effective, our findings do not suggest that clinician examiners are “impervious” to feedback about their severity, as has been suggested previously in the literature. On the contrary, they were interested in the feedback, and acknowledged the importance of being “on the same page” as other examiners. Examiner drift may be related in part to ongoing examiner attempts to calibrate their ratings. Calibration however appears to be difficult for examiners, and the impacts of examiner self-efficacy and examiner compliance may also complicate calibration. Much remains to be understood about clinical examiner judgments, including examiner severity, and examiner self-monitoring and meta-cognition. In the interim, we would argue that examiners should be provided with the most informative and useful data possible about their rating behaviours. The rating feedback in our own intervention may have been sub-optimal, and further research is indicated to explore optimal feedback strategies.
